# Helicobacter Pylori Infection in Waste Pickers: A Case Control Seroprevalence Study

**DOI:** 10.4021/gr578e

**Published:** 2013-10-31

**Authors:** Cosme Alvarado-Esquivel

**Affiliations:** a Biomedical Research Laboratory, Faculty of Medicine and Nutrition, Juarez University of Durango State, Avenida Universidad S/N, 34000 Durango, Dgo, Mexico

**Keywords:** *Helicobacter pylori*, Seroprevalence, Waste pickers, Epidemiology, Mexico

## Abstract

**Background:**

The epidemiology of *Helicobacter pylori* (*H. pylori*) infection in waste pickers had not been previously studied. This study aims to determine the association of *H. pylori* seropositivity and waste picking activity; and to determine socio-demographic, clinical, work, and behavioral characteristics associated with *H. pylori* seropositivity in waste pickers.

**Methods:**

Through a case-control study design, we examined 90 waste pickers and 90 age- and gender-matched control subjects for the presence of anti-*H. pylori* IgG antibodies using enzyme-linked immunoassays. Seroprevalence association with socio-demographic, clinical, work and behavioral characteristics of the waste pickers were also investigated.

**Results:**

Antibodies to *H. pylori* were found in 60 (66.7%) of the 90 waste pickers and in 51 (56.7%) of the 90 controls (P = 0.16). Stratification by age showed that waste pickers aged 14 -30 years old had significantly higher frequency of *H. pylori* infection than controls of the same age group (56.5% versus 35.6%, respectively; P = 0.04). The seroprevalence of *H. pylori* infection was not influenced by gender, age, educational level, socioeconomic status, residence, or housing conditions of waste pickers. The presence of underlying diseases and the frequency of gastritis were similar among *H. pylori* positive and *H. pylori* negative waste pickers. Logistic regression analysis showed that the duration (years) in the waste picking activity was positively associated with *H. pylori* exposure (OR = 2.76; 95% CI: 1.22 - 6.25; P = 0.01). In contrast, consumption of alcohol was negatively associated with *H. pylori* exposure (OR = 0.27; 95% CI: 0.09 - 0.78; P = 0.01). Other work or behavioral characteristics of waste pickers including washing hands before eating, eating from the garbage, animal contacts, consumption of unpasteurized milk, unwashed raw vegetables, fruits, or untreated water, and contact with soil were not associated with *H. pylori* exposure.

**Conclusions:**

This is the first report on the seroprevalence of *H. pylori* infection among waste pickers and the factors contributing to such exposure. Results warrant for further research on the potential role of contact with garbage for *H. pylori* infection.

## Introduction

*Helicobacter pylori* are microaerophilic Gram-negative bacterium currently infecting about one-half of the world’s population [1, 2]. Infections with *H. pylori* may be asymptomatic or may lead to gastric disease including chronic gastritis, peptic ulcer, gastric mucosa-associated lymphoid tissue lymphoma, and gastric cancer [1-4]. Transmission of *H. pylori* might occur from person-to-person [5], through oral-oral or oral-fecal routes [6], or consumption of contaminated water [6, 7]. Epidemiological studies indicate that prevalence of *H. pylori* infection varies substantially among countries and prevalence is mainly influenced by country development [8], geographical region, migration pattern and ethnicity [6, 9].

There is a lack of information about infections of *H. pylori* in waste pickers. Some epidemiological characteristics in waste pickers are relevant for acquiring infections with *H. pylori*. Firstly, the oral-oral and oral-fecal routes of *H. pylori* infection may occur in waste pickers. Garbage may contain human excrement, saliva and other fluids, therefore, waste pickers handle potentially contaminated garbage, and even a number of them eat from the garbage. Secondly, many waste pickers drink untreated water. Thirdly, the person-to-person route of infection with *H. pylori* might occur in waste pickers because they have poor living conditions including poor housing where a number of persons cohabitate in a single room. This case-control study aimed to determine the seroprevalence of anti-*H. pylori* antibodies in waste pickers in Durango City, Mexico. In addition, we also investigated socio-demographic, clinical, work, and behavioral characteristics associated with *H. pylori* seropositivity in waste pickers.

## Materials and Methods

### Study design and study population

Through an age- and gender- matched case-control study using serum samples from recent *Toxoplasma gondii* serosurveys [10, 11], 90 waste pickers and 90 control subjects were compared for the presence of anti-*H. pylori* IgG antibodies. Inclusion criteria for the waste pickers were: 1) waste pickers in the municipal solid waste transfer station of Durango City, Mexico; 2) aged 14 years and older; 3) any gender; 4) waste picking for at least 3 months; and 5) who accepted to participate in the study. Waste pickers included in the study were 14 - 76 (mean = 36.0 ± 17.1) year-old, 34 were males and 56 were females. Control subjects matched with waste pickers by age and gender. Controls consisted of 34 males and 56 females with miscellaneous occupations other than waste picking including students, employees, homemakers, and business. The mean age in controls was 35.7 ± 16.8 (range: 18 - 78) year-old and comparable with that in waste pickers (P = 0.91).

### Socio-demographic, clinical, work, and behavioral data

We obtained the characteristics of the participants by using a standardized questionnaire. Socio-demographic data including age, gender, birthplace, residence, educational level, and socioeconomic level was collected from all participants. Work characteristics assessed in waste pickers included number of years in the activity, habitual use of safety practices (use of hand gloves and face masks), eating while working, drinking alcohol while waste picking, washing hands before eating, eating from the garbage, and ever had suffered from injuries with sharp material of the garbage. Clinical data explored included the presence of underlying diseases in general and gastric disease in particular. Behavioral data included animal contacts, consumption of unpasteurized milk, unwashed raw vegetables, fruits, or untreated water, frequency of eating out of home (in restaurants or fast food outlets), and contact with soil (gardening or agriculture). Housing conditions were examined by using the Bronfman’s criteria [12]. Briefly, we evaluated five variables as follows: number of persons in the house, number of rooms in the house, material of the floor of the house, availability of drinkable water, and form of elimination of excreta.

### Serological detection of *H. pylori* antibodies

Serum samples from cases and controls were analyzed by qualitative and quantitative methods for detection of anti-*H. pylori* IgG antibodies using a commercially available enzyme-linked immunosorbent assay (ELISA) kit, Anti-*H. pylori* IgG AccuBind ELISA (Monobind Inc, Lake Forest, California). Anti-*H. pylori* IgG antibody levels were expressed as Units (U)/mL, and a value higher than 20 U/mL was considered a positive result. ELISA was performed following the manufacturer’s instructions.

### Statistical analysis

Statistical analysis was performed using the Epi Info version 3.5.4 software (Centers for Disease Control and Prevention: http://wwwn.cdc.gov/epiinfo/) and SPSS version 15.0 software (SPSS Inc. Chicago, Illinois). Pearson’s chi-square test and the Fisher exact test (when values were less than 5) were used to compare frequencies between groups. Bivariate and multivariate analyses were used to assess the association between waste pickers characteristics and *H. pylori* seropositivity. Variables were included in the multivariate analysis if they had a P value ≤ 0.20 in the bivariate analysis. Odd ratio (OR) and 95% confidence interval (CI) were calculated by multivariate analysis using logistic regression analysis with the Enter method. A P value < 0.05 was considered as statistically significant.

### Ethical considerations

This study was approved by the Ethical Committee of the Instituto de Seguridad y Servicios Sociales de los Trabajadores del Estado in Durango City. The purpose and procedures of the study were explained to all participants, and a written informed consent was obtained from each participant.

## Results

Of the 90 waste pickers tested, 60 (66.7%) were positive for anti-*H. pylori* IgG antibodies. While of the 90 controls tested, 51 (56.7%) were positive for anti-*H. pylori* IgG antibodies. In general, cases and controls had similar seroprevalences of anti-*H. pylori* IgG antibodies (P = 0.16). However, stratification by age showed the differences in the seroprevalence of *H. pylori* infection in cases and controls as follows. A 56.5 percent (26/46) versus 35.6% (16/45) in 14 - 30 years group (P = 0.04); 72.7% (16/22) versus 76% (19/25) in 31 - 50 years group (P = 0.79); and 80.1% (17/21) versus 80% (16/20) in 51 - 78 years group (*P*=0.62), respectively. General socio-demographic characteristics of the waste pickers studied shown in Table 1. The seroprevalence of *H. pylori* infection was not influenced by gender, age, residence, educational level, or socioeconomic status of waste pickers. Of the 60 *H. pylori* IgG positive waste pickers, 31 (51.7%) had IgG levels higher than 100 U/mL, 11 (18.3%) between 51 to 100 U/mL, and 18 (30.0%) between 21 to 50 U/mL. Anti-*H. pylori* IgG antibody levels were similar in men and women (P = 0.84). None of the housing variables examined including number of persons in the house, number of rooms in the house, material of the floor of the house, availability of drinkable water, and form of elimination of excreta showed any association with *H. pylori* infection.

**Table 1:** Socio-demographic Characteristics of Waste Pickers and Seroprevalence of *H. pylori* Infection

Characteristic	No. of subjects tested*	Prevalence of *H. pylori* infection		P value
		No.	%	
Gender				
Male	34	22	64.7	0.75
Female	56	38	67.9	
Age groups (years)				
30 or less	46	26	56.5	0.11
31 - 50	22	16	72.7	
> 50	21	17	80.0	
Residence place				
Durango State	88	58	65.9	0.55
Other Mexican State	2	2	100.0	
Residence area				
Urban	83	55	66.3	0.35
Suburban or rural	6	5	83.3	
Educational level				
No education	28	19	67.9	0.80
1 - 6 years	50	34	68.0	
More than 6 years	12	7	58.3	
Socioeconomic level				
Low	67	44	65.7	0.70
Medium	17	12	70.6	

* Participants with available data.

Of the work characteristics in waste pickers (Table 2), the years of duration in the activity and drinking alcohol while waste picking were positively and negatively associated with *H. pylori* infection, respectively. Other work characteristics including habitual use of safety practices, eating while working, washing hands before eating, eating from the garbage, and ever had suffered from injuries with sharp material of the garbage did not influence the seroprevalence of *H. pylori* infection.

**Table 2:** Work Characteristics of Waste Pickers and Seroprevalence of *H. pylori* infection

Characteristic	No. of subjects tested*	Prevalence of *H. pylori* infection		P value
		No.	%	
Years working				
Up to 10 years	47	26	55.3	0.03
11 to 20 years	29	21	72.4	
More than 20 years	13	12	92.3	
Number of visiting waste places				
One	80	53	66.3	0.67
More than 1	6	4	66.7	
Wear hand gloves				
Yes	16	10	62.5	0.64
No	73	50	68.5	
Wear facemask				
Yes	1	1	100.0	1.00
No	88	59	67.0	
Eating while working				
Yes	51	34	66.7	0.86
No	38	26	68.4	
Eating from the garbage				
Yes	26	15	57.7	0.20
No	63	45	71.4	
Washing hands before eating				
Yes	68	45	66.2	0.65
No	21	15	71.4	
Alcohol consumption				
Yes	29	15	51.7	0.02
No	60	45	75.0	
Injury while working				
Yes	64	40	62.5	0.13
No	24	19	79.2	

* Participants with available data.

With respect to clinical data, the presence of underlying diseases and the frequency of gastritis were similar among *H. pylori* positive and *H. pylori* negative waste pickers.

Concerning behavioral characteristics, bivariate analysis showed 2 characteristics with a P value equal to or less than 0.20 including: cats at home (P = 0.13), and raising animals (P = 0.17). Other behavioral characteristics including consumption of unpasteurized milk, unwashed raw vegetables, fruits, or untreated water, frequency of eating out of home and contact with soil showed P values higher than 0.20 in the bivariate analysis. Multivariate analysis (Table 3) of the work and behavioral characteristics with a P value equal to or less than 0.20 obtained by bivariate analysis showed that the years of duration in the waste picking activity was positively associated with *H. pylori* exposure (OR = 2.76; 95% CI: 1.22 - 6.25; P = 0.01). In contrast, consumption of alcohol was negatively associated with *H. pylori* exposure (OR = 0.27; 95% CI: 0.09 - 0.78; P = 0.01). No further work or behavioral characteristics of waste pickers associated with *H. pylori* exposure were found.

**Table 3:** Results of the multivariate regression analysis.

Variable	P value	Odds ratio	95% confidence interval
Cats at home	0.2	1.88	0.70 - 5.05
Raising animals	0.68	1.23	0.44 - 3.42
Alcohol consumption	0.01	0.27	0.09 - 0.78
Years in the activity	0.01	2.76	1.22 - 6.25

## Discussion

In the present study, waste pickers showed a slightly higher (but not statistically significant) seroprevalence of *H. pylori* infection than age- and gender-matched control subjects. The general seroprevalence found in waste pickers is comparable to the mean national seroprevalence (66%) reported in Mexico [13]. However, these seroprevalence results cannot be directly compared, since different methods were used in both studies; although the present study used a commercial ELISA kit, a homemade ELISA kit was used for the national survey. In an international context, the rate of *H. pylori* seroprevalence in the waste pickers of Durango is lower than the estimated 80-90% seroprevalence of *H. pylori* infection in developing countries [8]. In the present study, further analysis by age stratification showed that waste pickers aged 14 - 30 years old had a significantly higher seroprevalence of *H. pylori* infection than controls of the same age group. While in older age groups of waste pickers and controls, the seroprevalence of *H. pylori* infection was comparable. These findings suggest that waste picking may be an important factor for *H. pylori* exposure in young subjects but there might be unidentified behavioral, work, or other type of changes leading to a similar frequency of *H. pylori* exposure in both waste pickers and controls of older ages. Therefore, results suggest that young waste pickers represent a risk group for *H. pylori* infection. Of note, the 56.5% seroprevalence of *H. pylori* infection found in young (≤ 30 years old) waste pickers is also higher than the 37.9% seroprevalence reported recently in Mennonites of the same age group in a rural community in Durango State [14]. Remarkably, analysis of waste pickers socio-demographic characteristics showed that seroprevalence of *H. pylori* was not only high among the youngest participants but also did not show a statistically significant increase with age. The former suggests an early exposure to *H. pylori* in waste pickers, and the latter is not consistent with the typical increase in the frequency of infection with age that has been reported in several studies [5, 9, 14-16]. Other putative socio-demographic factors associated with *H. pylori* infection, including low socioeconomic status [5, 13] and low educational level [13], did not influence seroprevalence rates in waste pickers. Similarly, *H. pylori* seropositivity was not associated with gender or residence. Concerning housing conditions of waste pickers, seroprevalence of *H. pylori* was not influenced by the number of persons in the house, number of rooms in the house, material of the floor of the house, availability of drinkable water, and form of elimination of excreta. These findings suggest that *H. pylori* exposure might have not occurred at home by drinking untreated water, overcrowding or poor sanitary conditions but probably in the waste transfer station by strong contact with garbage likely contaminated with human excrement and fluids.

Concerning clinical data, the presence of underlying diseases and the frequency of gastritis were similar among *H. pylori* positive and *H. pylori* negative waste pickers. This finding suggests that *H. pylori* are not probably influencing on the health of waste pickers.

With respect to work and behavioral characteristics of waste pickers, multivariate analysis showed that the years of duration in the waste picking activity was positively associated with *H. pylori* exposure (OR = 2.76; 95% CI: 1.22 - 6.25; P = 0.01). In contrast, consumption of alcohol was negatively associated with *H. pylori* exposure (OR = 0.27; 95% CI: 0.09 - 0.78; P = 0.01). No further work or behavioral characteristics of waste pickers associated with *H. pylori* exposure were found. The association of *H. pylori* seropositivity and the years of duration in the waste picking activity further support the increased risk for *H. pylori* infection by waste contact. It is not clear how the waste pickers acquired the *H. pylori* infection but it is likely that they acquired it through frequent contact with garbage contaminated with human excrements and fluids. The negative association of alcohol consumption with *H. pylori* seropositivity found in the current study supports previous observations [17-20] and suggests that alcohol consumption might be protective against *H. pylori* infection. However, the negative association of alcohol consumption and *H. pylori* infection conflicts with observations in other studies. Zhang et al [21] reported a positive association of alcohol consumption with *H. pylori* infection in patients with functional dyspepsia. In contrast, no association of alcohol consumption and *H. pylori* infection have been found in blood donors in Brazil [22], and in patients with abdominal complains in a clinic in Japan [23]. Differences in the characteristics of the studied populations including health status and occupation may explain the differences in the associations.

This is the first report on the seroprevalence of *H. pylori* infection among waste pickers and the factors contributing to such exposure. Results warrant for further research on the potential role of contact with garbage for *H. pylori* infection.
